# Inhibition of HAS2 and hyaluronic acid production by 1,25-Dihydroxyvitamin D_3_ in breast cancer

**DOI:** 10.18632/oncotarget.27587

**Published:** 2020-07-28

**Authors:** Carmen J. Narvaez, Erika LaPorta, Samantha Robilotto, Jennifer Liang, JoEllen Welsh

**Affiliations:** ^1^University at Albany Cancer Research Center, Rensselaer, NY, USA; ^2^Department of Environmental Health Sciences, University at Albany, Rensselaer, NY, USA; ^3^Department of Biomedical Sciences, University at Albany, Rensselaer, NY, USA; ^4^Department of Biochemistry, Queen’s University, Kingston, ON, Canada; ^*^Joint first authors

**Keywords:** vitamin D, hyaluronic acid, hyaluronan synthase, HAS2, breast cancer

## Abstract

1,25-Dihydroxyvitamin D_3_ (1,25D3) induces growth arrest and apoptosis in breast cancer cells *in vivo* and *in vitro*, however the exact mechanisms are unclear. Although the vitamin D receptor (VDR), a ligand dependent transcription factor, is required for growth regulation by vitamin D, the specific target genes that trigger these effects are unknown. Genomic profiling of murine mammary tumor cells with differential VDR expression identified 35 transcripts that were altered by the 1,25D3-VDR complex including Hyaluronan Synthase-2 (*Has2*). Here we confirmed that 1,25D3 reduces both *HAS2* gene expression and hyaluronic acid (HA) synthesis in multiple models of breast cancer. Furthermore, we show that the growth inhibitory effects of 1,25D3 are partially reversed in the presence of high molecular weight HA. *HAS2* expression and HA production are elevated in immortalized human mammary epithelial cells induced to undergo epithelial-mesenchymal transition (EMT) through stable expression of TGFβ, SNAIL or TWIST and in those expressing oncogenic H-RAS^V12^, indicating that deregulation of HA production may be an early and frequent event in breast tumorigenesis. 1,25D3 also reduces HA secretion and acts additively with an HA synthesis inhibitor to slow growth of cells expressing TGFβ, SNAIL and TWIST. Analysis of mammary gland and tumors from *Vdr* knockout mice suggest that loss of VDR is associated with enhanced HAS2 expression and HA production *in vivo*. These data define a novel role for 1,25D3 and the VDR in control of HA synthesis in epithelial tissues that likely contributes to its anti-cancer actions.

## INTRODUCTION

1,25-Dihydroxyvitamin D_3_ (1,25D3), the high affinity ligand for the nuclear VDR, regulates multiple cancer processes (cell cycle, apoptosis, migration, invasion) *in vivo* and *in vitro*, however the specific gene targets and mechanisms that mediate these effects are unclear. We previously established invasive mammary tumor cell lines from wild-type (WT) and VDR knockout (KO) mice and demonstrated that the VDR is necessary for 1,25D3 mediated anti-cancer signaling *in vitro* and *in vivo* [1–3]. Genomic profiling in this model system [4, 5] identified 35 transcripts that were altered by the 1,25D3-VDR complex, only four of which were down-regulated. One of the VDR down-regulated genes was *Has2* (hyaluronan synthase-2), an enzyme that synthesizes the polysaccharide hyaluronic acid (HA). 1,25D3 treatment (100 nM, 24 h) reduced *Has2* expression 50–70% in VDR positive cells but was without effect in VDR negative cells. *Has2* encodes one of three integral membrane proteins (*Has1*, *Has2*, and *Has3*) that synthesize and extrude HA into the pericellular space [6]. Mature HA is a heterogeneous polysaccharide (MW ranges from 5,000 to 20,000,000 Da *in vivo*) that is ubiquitously present in normal tissues. However, considerable evidence indicates that virtually all human epithelial tumors exhibit elevated amounts of HA and that tumor HA content negatively correlates with disease progression and survival [7–12]. HA is a linear chain of glucuronic acid and N-acetyl-D-glucosamine units which are derived from the hexosamine biosynthesis pathway. High activity of the hexosamine pathway in breast cancer cells leads to HA accumulation and pro-tumorigenic signaling, making this pathway a relevant therapeutic target [13]. HAS2 and HA promote epithelial-mesenchymal transition (EMT), survival, invasion and metastasis *in vitro* and *in vivo* [14–21]. Many of these effects result from HA-mediated activation of CD44, a pro-survival receptor enriched on the surface of cancer stem cells [17, 22–26]. Collectively, these data suggest that survival and outgrowth of CD44+ cancer stem cells are dependent on continued HA synthesis through HAS2 activity. This concept predicts that disruption of HA-CD44 signaling would inhibit disease progression in patients whose tumors overexpress *HAS2*. In the studies reported here we assessed whether 1,25D3 regulates *HAS2* in cellular models of human breast cancer, and whether suppression of *HAS2* by 1,25D3 is sufficient to inhibit HA synthesis in the context of aggressive disease.

## RESULTS

### *Has2* mRNA is down-regulated by 1,25D3 in murine mammary carcinoma cells

**Figure 1:**
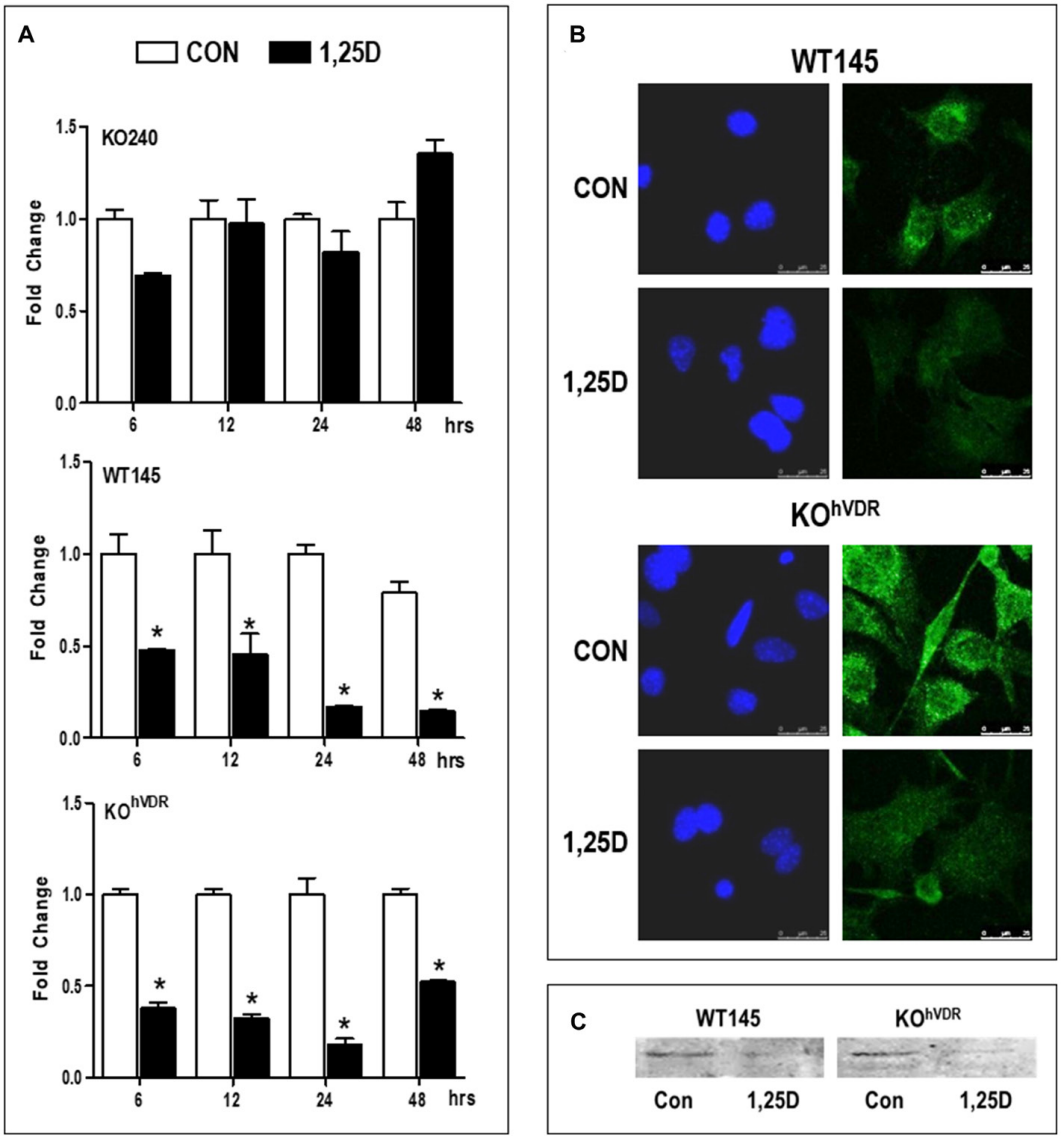
VDR is required for 1,25D3 mediated down-regulation of *Has2* mRNA and protein. (**A**) RNA was isolated from KO240, WT145 and KO^hVDR^ cells treated with 100 nM 1,25D3 for 6, 12, 24, or 48 hours. *Has2* mRNA in control and 1,25D3 treated samples was assessed by the ΔΔC_t_ method and values were normalized against *18S* and expressed as fold change (1,25D3 *vs* control) for each cell line. Bars represent mean ± standard deviation, ^*^ *p* < 0.05 control *vs* 1,25D3 treated at each time point as evaluated by Student’s *t* test. (**B**) Immunofluorescence for HAS2 (green) in WT145 and KO^hVDR^ cells treated with 100 nM 1,25D3 or vehicle for 48 hours. Nuclei were stained with DAPI (blue). Images were acquired on a Leica DMI6000 microscope with a TCS SP5 confocal laser scanner using Leica Application Suite software. (**C**) Lysates from WT145 and KO^hVDR^ cells treated with 100 nM 1,25D3 for 48 hours were blotted with antibodies against HAS2.

In previous studies we demonstrated that 1,25D3 down-regulated mRNA expression of the HA synthesizing enzyme *Has2* in a VDR-dependent manner after 24 hours [4]. Here we have extended these findings to assess whether regulation of *Has2* mRNA by 1,25D3 alters HA production and/or phenotype of breast cancer cells. We first examined the kinetics of *Has2* mRNA down-regulation by 1,25D3 in KO240, WT145, and KO^hVDR^ cells. RT-qPCR was conducted in samples harvested 6, 12, 24, and 48 hours after treatment with 100 nM 1,25D3 or vehicle (Figure 1A). In KO240 cells lacking VDR, *Has2* mRNA was variable with up and down trends over the time course and no consistent effect of 1,25D3. In contrast, 1,25D3 reduced *Has2* expression at all time points tested in WT145 cells (which express murine *Vdr*) and in KO^hVDR^ cells (KO240 cells engineered to ectopically express human VDR). Both VDR positive cell lines displayed down-regulation of *Has2* within 6 hours of 1,25D3 treatment, with the peak decrease (approximately 25% of control values) at 24 hours and suppression sustained through 48 hours.

We assessed HAS2 protein expression by immunofluorescent staining of WT145 and KO^hVDR^ cells that were treated with 1,25D3 or vehicle for 48 hours. As shown in Figure 1B, confocal imaging localized punctate staining of HAS2 on cell surfaces, and treatment with 1,25D3 reduced staining intensity in both WT145 and KO^hVDR^ cells. Western blotting confirmed down-regulation of HAS2 protein in VDR positive cells treated with 1,25D3 for 48 h (Figure 1C). Collectively, these data demonstrate that the down-regulation of *Has2* mRNA by 1,25D3 requires VDR and is of sufficient magnitude to reduce HAS2 protein expression.

### 1,25D3 reduces cell-associated and secreted HA

**Figure 2:**
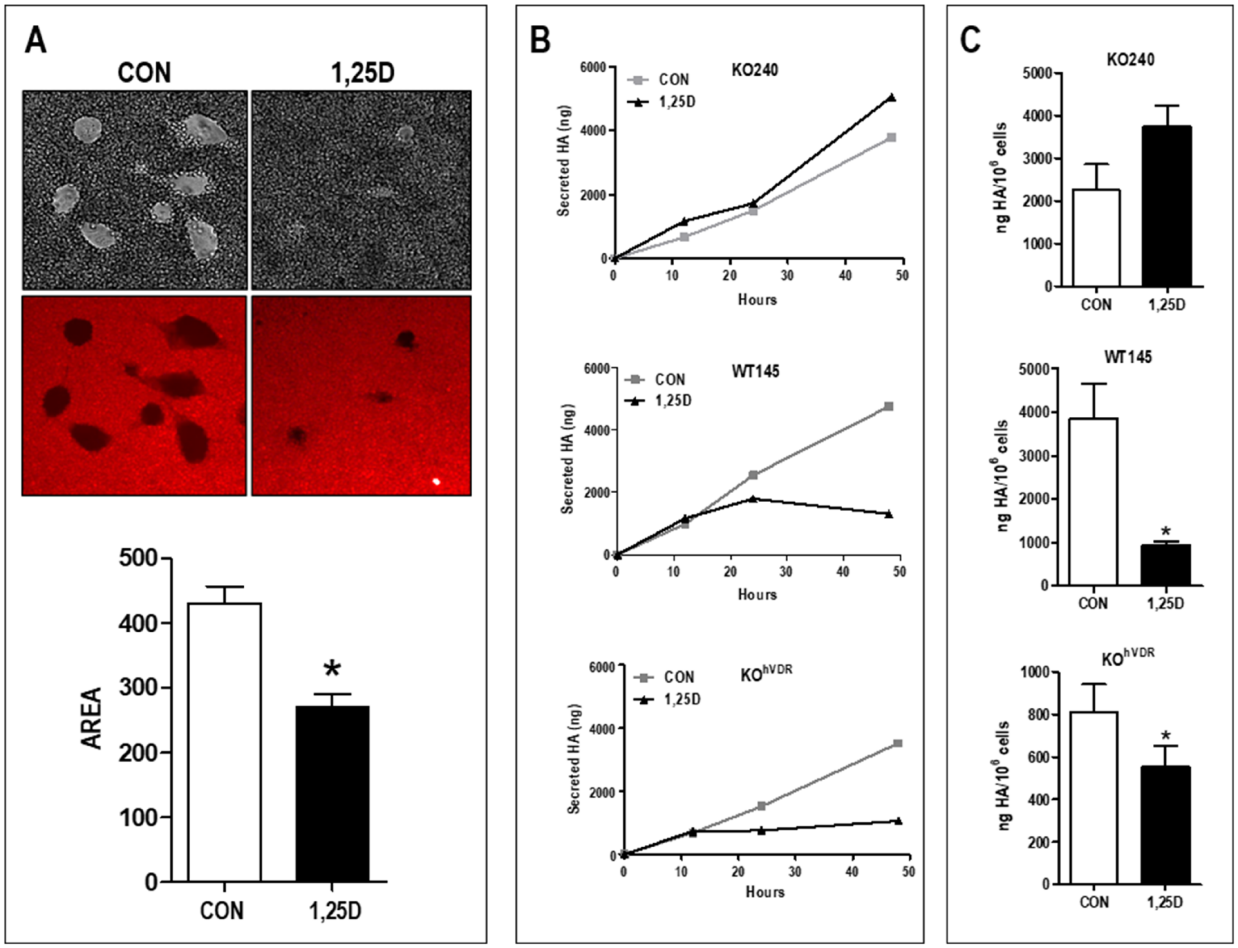
1,25D3 reduces both cell surface and secreted HA. (**A**) Cell associated HA was imaged by particle exclusion assay in which sheep erythrocytes are overlaid onto cell monolayers. Shown are KO^hVDR^ cells treated for 48 hours with vehicle or 100 nM 1,25D3 prior to assay. Areas in which erythrocytes (captured as autofluorescence in the FITC channel) are excluded represent the HA-rich pericellular matrix. Graph depicts quantitation of the exclusion area in 19–21 fields for each treatment (mean ± standard error, ^*^ *p* < 0.0001; Student’s *t* test). (**B**) Secreted HA was quantitated by ELISA of conditioned media of KO240, WT145 and KO^hVDR^ cells sampled at 0, 12, 24 or 48 hours after treatment with 100 nM 1,25D3 or vehicle. (**C**) Total secreted HA after 72 hours normalized to final cell number (mean ± standard error, ^*^ *p* < 0.05; Student’s paired *t* test).

To examine if the reduction of *Has2* in response to 1,25D3 treatment translated to a reduction in HA production, we assessed both cell-associated and secreted HA. In the majority of cell types, newly synthesized HA is extruded at the plasma membrane and associates with cell surface proteins, forming an extensive pericellular coat. This pericellular matrix can be imaged by particle exclusion assays which employ red blood cells that are repelled by HA [27]. As shown in Figure 2A, distinct exclusion areas surround control KO^hVDR^ cells, allowing visualization of the HA-coated cell surfaces. In contrast, the surfaces of 1,25D3 treated KO^hVDR^ cells are barely visible, indicating the absence of an HA-rich pericellular coat. To quantitate HA synthesis, conditioned media samples were collected from KO240, WT145, and KO^hVDR^ cells at time 0 and 12, 24, and 48 hours after treatment with 100 nM 1,25D3. Total HA was evaluated with a solid-phase sandwich ELISA employing biotinylated HA binding protein (bHABP) which detects both low and high MW HA chains. As shown in Figure 2B, all three cell lines secreted HA which accumulated in media over time. However, only VDR positive cells (WT145 and KO^hVDR^) exhibited a decrease in HA accumulation in response to 1,25D3 treatment. The amount of secreted HA normalized for final cell numbers at 72 hours was significantly reduced in VDR positive cells but not in VDRKO cells (Figure 2C). These data are consistent with the observed VDR dependent effects of 1,25D3 on *Has2* mRNA expression (Figure 1) and indicate that *Has2* down-regulation by 1,25D3 is of sufficient magnitude to disrupt HA synthesis and secretion.

### HA rescues cells from 1,25D3 mediated growth inhibition

**Figure 3:**
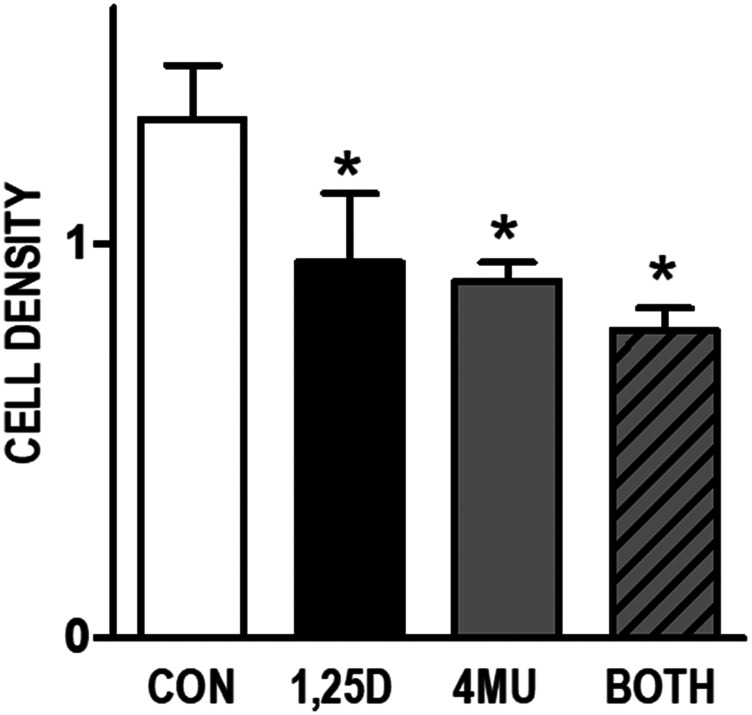
Effects of HA synthesis inhibitor 4MU and 1,25D3 on growth of WT145 cultures. WT145 cells were treated with 500 μM 4-methylumberellifone (4MU) in the presence or absence of 100 nM 1,25D3 for 96 hours. Adherent cell growth was measured by crystal violet absorbance. Bars represent mean ± standard error of at least 2 independent samples analyzed in quadruplicate. *p* < 0.05, control *vs* treatments as assessed by one-way ANOVA and Bonferroni’s Multiple Comparison test.

**Figure 4:**
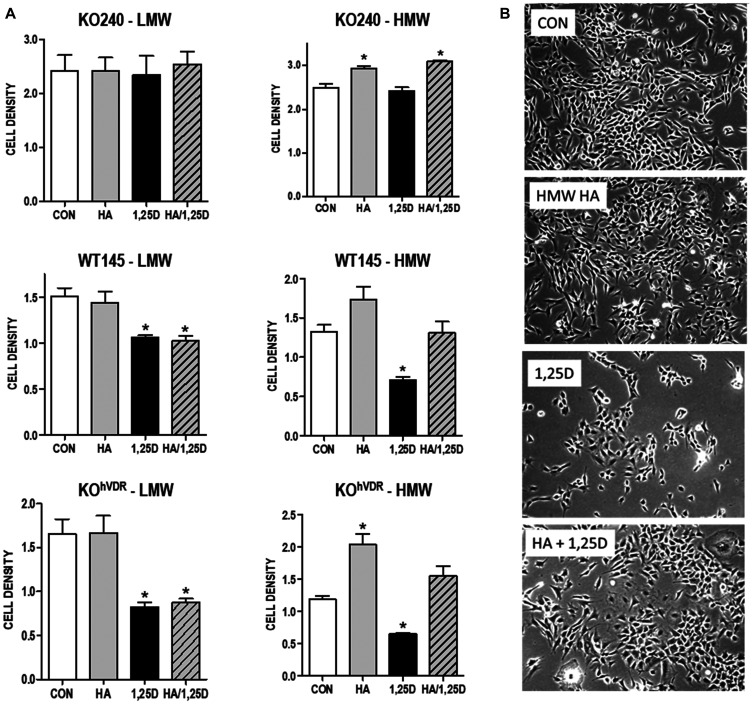
Effect of exogenous HA ± 1,25D3 on growth and morphology of mammary tumor cells. (**A**) KO240, WT145 and KO^hVDR^ cells were treated with 500 mg/mL high molecular weight (HMW) or low molecular weight (LMW) HA in the presence or absence of 100 nM 1,25D3 for 96 hours. Adherent cell growth was measured by crystal violet absorbance. Bars represent mean ± standard error of 2 independent experiments analyzed in quadruplicate. ^*^ *p* < 0.05, control *vs* individual treatments assessed by one-way ANOVA. (**B**) Phase contrast images of WT145 cells 96 hours after treatment with vehicle, 500 mg/mL HMW HA, 100 nM 1,25D3 or the combination of 1,25D3 and HA.

To assess the contribution of HA to cell growth in this model system, we first examined the effect of the HA synthesis inhibitor 4-methylumbelliferone (4MU) on cell density. 4MU, which binds UDP-glucuronic acid and prevents its incorporation into HA [28], significantly reduced the amount of HA secreted from WT145 cells (Supplementary Figure 1). Since 4MU does not block HA synthesis via targeting *Has2* expression, we anticipated that combining 4MU with 1,25D3 would additively inhibit cell growth. In WT145 cells, both 1,25D3 (100 nM) and 4MU (500 μM) inhibited culture density, and density was lowest in cultures treated simultaneously with 1,25D3 and 4MU (Figure 3). We next examined whether exogenous addition of HA could rescue 1,25D3 treated cells from growth inhibition. Because HA can exert unique effects depending on molecular size, we tested both High Molecular Weight HA (HMW, 1.54 × 10^6^ kDa) and Low Molecular Weight HA (LMW, 29 kDa) in these assays. Cell density was assessed in KO240, WT145, and KO^hVDR^ cells treated for 96 hours with 100 nM 1,25D3, 500 μg/mL HA (LMW or HMW), the combination of both, or vehicle. As shown in Figure 4A, growth of all three cell lines was unaffected by LMW HA but was increased by HMW HA. As expected, the effects of HA on KO240 cells lacking VDR were not altered in the presence of 1,25D3. However, growth of both WT145 and KO^hVDR^ cells was inhibited by 1,25D3 and co-treatment with HMW HA (but not LMW HA) abrogated this growth inhibition. The morphology of WT145 cells after 96 h treatment with 1,25D3 in the presence or absence of HMW HA is shown in Figure 4B. We observed a clear increase in cell density with no change in overall morphology in cultures treated with 1,25D3 and HMW HA relative to those treated with 1,25D3 alone. These data suggest that HMW HA can rescue 1,25D3 mediated growth inhibition, supporting the concept that suppression of *Has2* and HA synthesis contributes to VDR dependent growth regulation.

### Effect of 1,25D3 on additional genes involved in HA synthesis and degradation

Having determined that 1,25D3 down-regulates expression of *Has2* mRNA in a VDR dependent manner, genes coding for the remaining two HA synthesizing enzymes (*Has1* and *Has3*) and three enzymes involved in HA degradation (*Hyal1*, *Hyal2*, and *Hyal3*) were selected for further analysis. RT-qPCR was performed on WT145 and KO^hVDR^ cells treated with 100 nM 1,25D3 or ethanol vehicle for 24 hours. *Has1* and *Has3* expression were not altered by 1,25D3 treatment in WT145 or KO^hVDR^ cells (Supplementary Figure 2). Similarly, 1,25D3 did not significantly alter *Hyal1* or *Hyal3* expression in WT145 or KO^hVDR^ cells. 1,25D3 treated KO^hVDR^ cells displayed a non-significant increase in *Hyal2* expression compared to vehicle treated cells. Thus, of the genes involved in HA synthesis or degradation that were analyzed, only *Has2* is consistently regulated by 1,25D3/VDR signaling. Similar results were observed in tumor associated fibroblasts isolated from WT mice (Supplementary Figure 3), with the exception that 1,25D3 up-regulated *Hyal3* in fibroblasts. No effects of 1,25D3 were observed in tumor associated fibroblasts isolated from VDRKO mice (not shown). These data suggest that VDR dependent suppression of *Has2* by 1,25D3 may extend to the tumor microenvironment.

### HAS2 and the HA pathway is up-regulated in tissues from VDRKO mice

**Figure 5:**
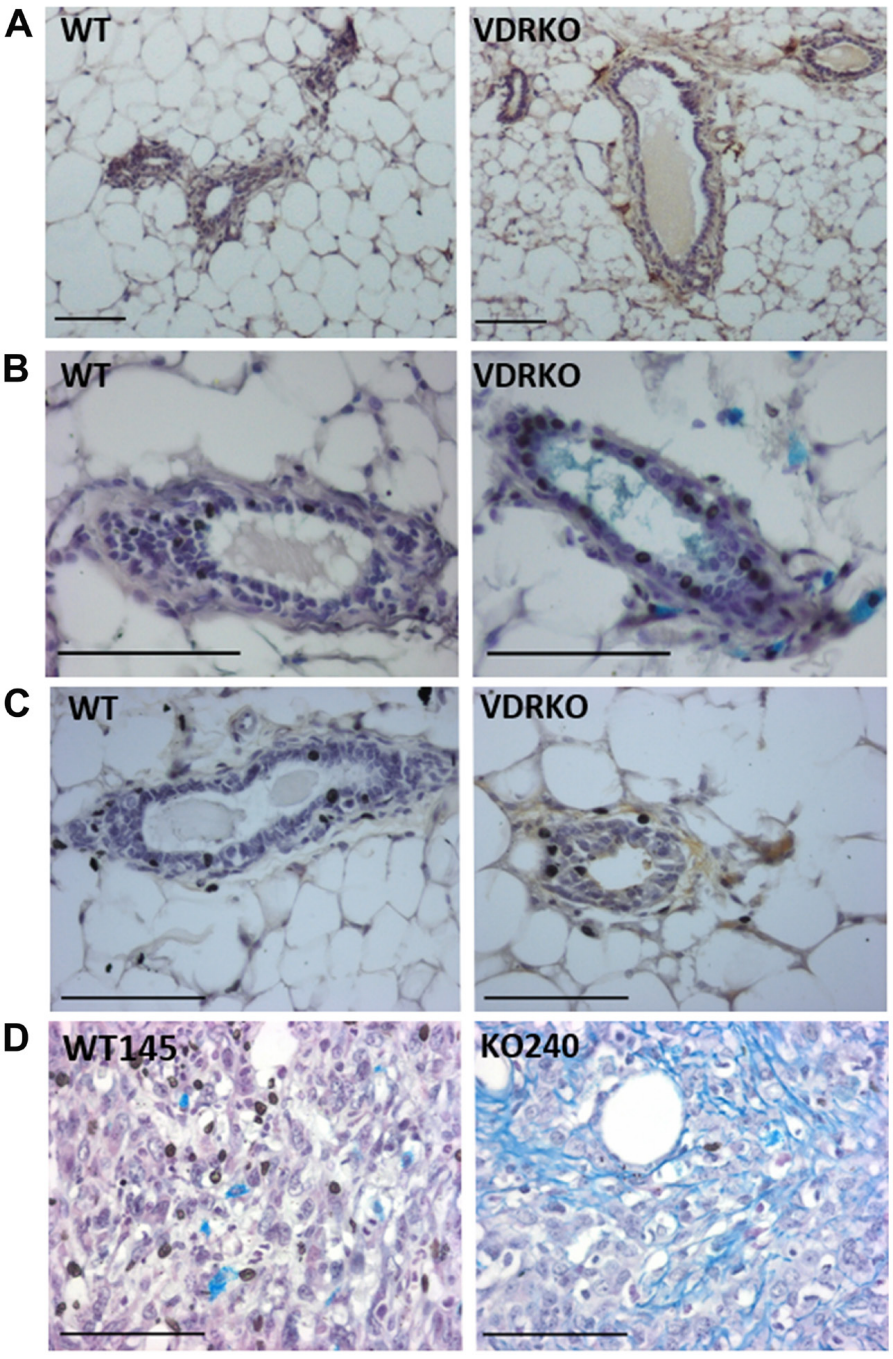
HAS2, glycosaminoglycans and HA localization in wild-type and VDRKO mice. (**A**) Mammary glands from female wild-type (WT) and VDRKO mice maintained on high calcium rescue diet were formalin fixed, paraffin embedded and processed for immunohistochemistry with HAS2 antibody. Brown staining indicates HAS2 positivity. (**B**) Glycosaminoglycans including HA were detected by alcian blue staining of glandular tissue from WT and VDRKO mice. (**C**) HA was localized by bHABP and detected with DAB chromogen in archived glandular tissue [30]. (**D**). Alcian blue staining of xenografts composed of WT145 and KO240 cell lines [1].

In previous studies we demonstrated that ablation of *Vdr* alters mammary gland development and tumorigenesis [29, 30]. Using archived paraffin embedded sections from those previous studies, we assessed HAS2 and HA abundance in tissues from adult female WT and VDRKO mice. Very few HAS2 positive cells were identified in mammary tissue of WT mice, whereas HAS2 positive cells were frequent in the stroma of glands from VDRKO mice (Figure 5A). Similarly, Alcian blue staining for glycosaminoglycans was minimal in WT tissue but prominent in stroma surrounding mammary ducts of VDRKO mice (Figure 5B). Specific localization of HA with bHABP also indicated stronger staining around and within ducts of glandular tissue from VDRKO mice relative to WT mice (Figure 5C). Similar results were observed in skin of VDRKO mice (Supplementary Figure 5) which also displays enhanced susceptibility to carcinogenesis relative to WT mice [31]. In xenografts composed of *Vdr* positive WT145 cells, Alcian blue staining presented as small clusters between tumor cells, whereas in xenografts of KO240 cells staining was more extensive, with long filamentous strands visible in close association with tumor cells (Figure 5D). Collectively, these data support the concept that VDR suppresses HAS2 and HA production in both normal mammary gland and in tumor cells *in vivo*.

### HA pathway is elevated during human mammary epithelial cell transformation

**Figure 6:**
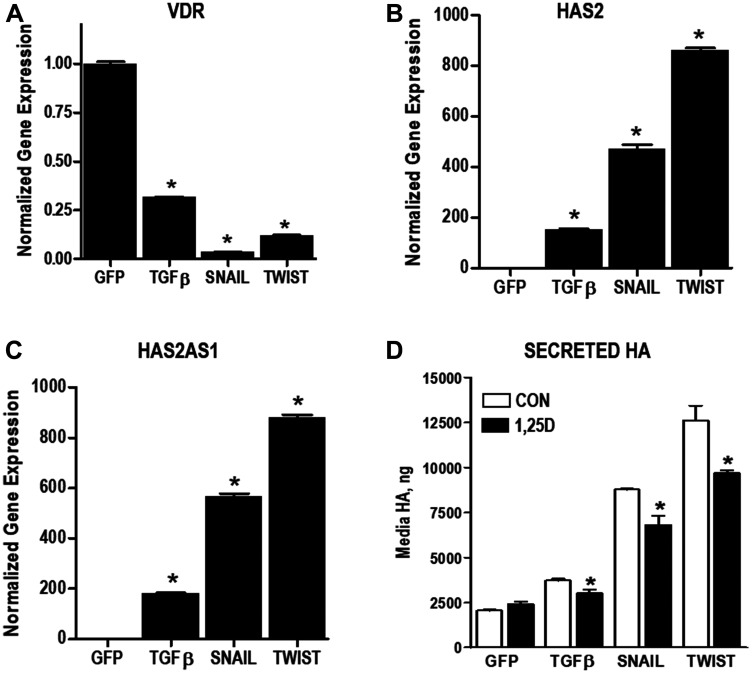
Changes in VDR and HA pathway during EMT. (**A**–**C**) RNA was isolated from immortalized human mammary epithelial (HMLE) cells stably expressing GFP (control), TGFβ, SNAIL or TWIST. Expression of *VDR* (A), *HAS2* (B) and *HAS2AS1*(C) were evaluated by RT-qPCR, normalized to 18S expression and expressed relative to GFP control cell line. Bars represent mean ± standard deviation of at 3 independent samples analyzed in triplicate. ^*^ *p* < 0.05 *vs* expression in HMLE cells as determined by one-way ANOVA and Dunnett’s post hoc test. (**D**) Secreted HA was evaluated in conditioned media of HMLE cells stably expressing GFP, TGFβ, SNAIL or TWIST by ELISA. ^*^ *p* < 0.05, 1,25D3 treated *vs* control for each cell line as evaluated by Student’s *t* test.

**Figure 7:**
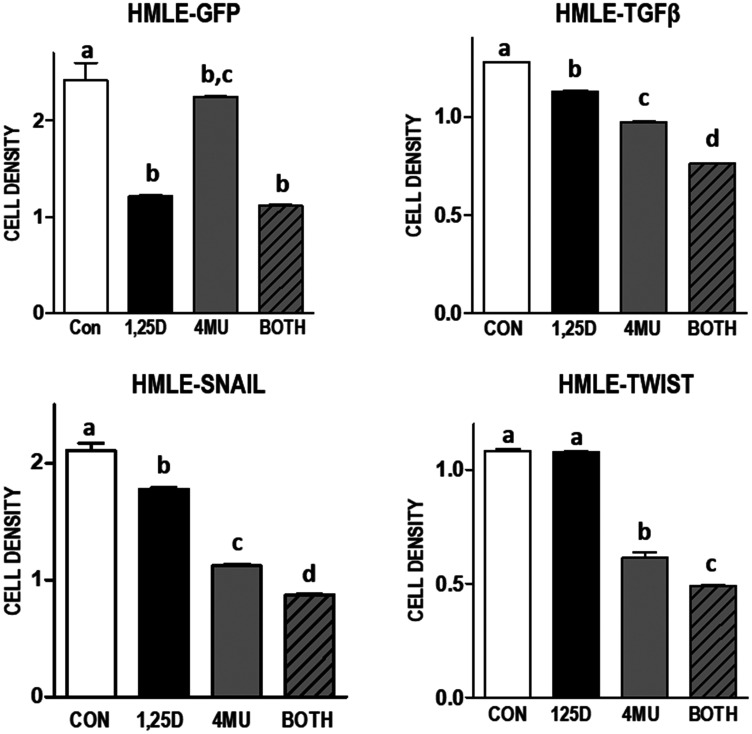
Effect of 1,25D3 and HA synthesis inhibitor 4MU on culture density in mammary epithelial cell models of EMT. Indicated cell lines were treated with 100 μM 4-methylumberellifone (4MU) in the presence or absence of 100 nM 1,25D3 for 96 hours. Adherent cell growth was measured by crystal violet absorbance and expressed relative to untreated samples for each cell line. Data are mean ± standard deviation, *n* = 6. Bars annotated with different letters are significant at *p* < 0.05 by one-way ANOVA and Bonferroni post -test.

Although many studies have demonstrated elevated HA in aggressive tumors, little is known about changes in the HA pathway during tumor development. We used a well characterized model of EMT [32] to assess whether the HA pathway is altered during this stage of carcinogenesis. VDR and HA related gene expression was compared in immortalized human mammary epithelial cells (HMLE) expressing GFP (control cells) to HMLE cells expressing factors known to induce EMT (TGFβ, SNAIL or TWIST). As shown in Figure 6, mRNA expression of *VDR* is dramatically reduced, while *HAS2* is strongly increased, in cells that have undergone EMT regardless of the trigger (TGFβ, SNAIL, or TWIST). In addition, mRNA for *HAS2-AS1,* the natural antisense transcript of *HAS2* which induces transcription of the *HAS2* gene [33], was increased in parallel with *HAS2* mRNA during EMT. Concomitant with the changes in *HAS2* and *HAS2-AS1* expression, secretion of HA into conditioned media was significantly elevated in TGFβ, SNAIL and TWIST expressing cells. 1,25D3 suppressed HA secretion from TGFβ, SNAIL, and TWIST expressing cells suggesting residual 1,25D3 signaling despite VDR down-regulation. In support of an important role for HA in sustaining EMT, TGFβ, SNAIL, and TWIST expressing cells were more sensitive to 4MU mediated growth inhibition than parental (GFP) cells (Figure 7). In addition, 1,25D3 cooperated with 4MU to inhibit growth of cells that had undergone EMT but not that of control cells.

**Figure 8:**
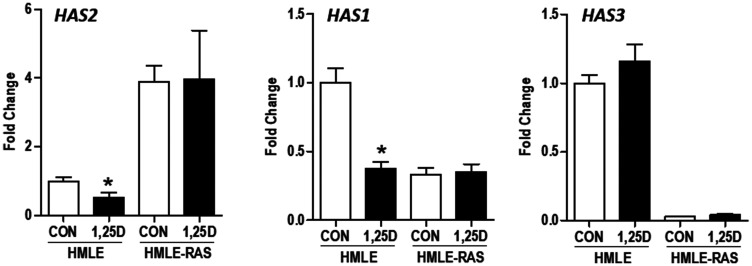
Effect of H-RAS^V12^ transformation on *HAS* gene expression in immortalized human mammary epithelial cells. RNA was isolated from control human mammary epithelial (HMLE) cells and those expressing oncogenic H-RAS^V12^ (HMLE-RAS) 24 hours after treatment with 100 nM 1,25D3 or vehicle. *HAS2*, *HAS1*, and *HAS3* expression was evaluated by RT-qPCR, normalized to 18S mRNA and expressed relative to untreated HMLE cells. Bars represent mean ± standard deviation of 3 independent samples analyzed in quadruplicate. ^*^ *p* < 0.05, 1,25D3 treated *vs* control in each cell line as evaluated by Student’s *t* test.

In a second model of HMLE transformation we assessed the impact of H-RAS^V12^, an oncogenic form of RAS, on the HA pathway in the presence and absence of 1,25D3. In non-transformed HMLE cells, 1,25D3 significantly suppressed *HAS2* and *HAS1* mRNA expression but was without effect on *HAS3* (Figure 8). Upon H-RAS^V12^ transformation, *HAS2* was significantly increased while both *HAS1* and *HAS3* were strongly reduced. Consistent with previous reports that RAS transformation abrogates VDR signaling [34–36], 1,25D3 failed to suppress *HAS2* mRNA in HMLE-RAS cells and did not alter the expression of *HAS1* or *HAS3*. Of the hyaluronidase genes, *HYAL1* was increased while *HYAL2* was decreased in HMLE-RAS cells relative to HMLE parental cells, but no consistent effects of 1,25D3 on these genes were noted (data not shown).

### 1,25D3 reduces *HAS2* and HA in human breast cancer cells

**Figure 9:**
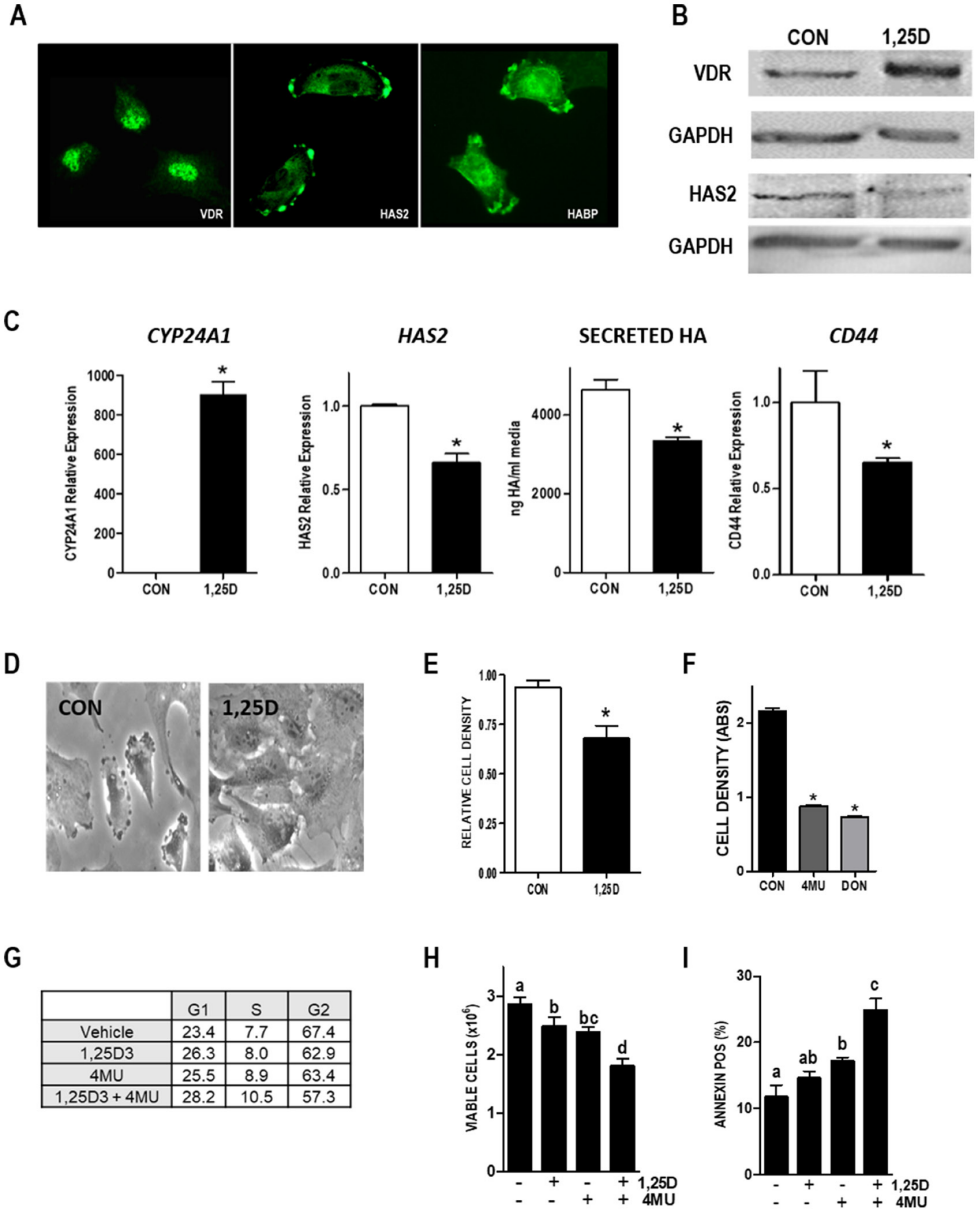
Vitamin D and HA pathways in Hs578T human TNBC cells. (**A**) Images of VDR, HAS2, and HA localization in Hs578T cells. (**B**) Lysates from Hs578T cells treated with vehicle or 100 nM 1,25D3 for 48 hours were blotted with antibodies against VDR, HAS2, or loading control GAPDH. (**C**) RNA was isolated from Hs578T cells treated with 100 nM 1,25D3 for 24 hours. *CYP24A1*, *HAS2* and *CD44* were quantitated by RT-qPCR, normalized to 18S and expressed relative to vehicle treated cells. Secreted HA was evaluated by ELISA of conditioned media removed 48 hours after 1,25D3 or vehicle treatment. Bars represent mean ± standard error, with quadruplicates for RT-qPCR and triplicates for ELISA. ^*^ *p* < 0.05, control *vs* 1,25D3 treated in each cell line as evaluated by Student’s *t* test. (**D**) Phase contrast images of Hs578T cells treated with 100 nM 1,25D3 or vehicle for 96 hours. (**E**) Crystal violet assay for culture density was conducted in HS578T cells treated with 100 nM 1,25D3 for 144 hours. (**F**) Crystal violet assay for culture density was conducted in HS578T cells treated with HA synthesis inhibitors 4MU or DON for 96 hours. Data from growth assays are mean ± standard error of quadruplicates. ^*^ *p* < 0.05, control *vs* treated as evaluated by Student’s *t* test. (**G**) Percentages of cells in G1, S and G2 phases of the cycle after 96 hours treatment with 100 nM 1,25D ± 100 μM 4MU. Data are mean of duplicates with 5000 cells analyzed per run. (**H**) Viable cell count (mean ± standard deviation, *n* = 4) after 96 hours treatment with 100 nM 1,25D ± 100 μM 4MU. Bars annotated with different letters are significant at *p* < 0.05 by one-way ANOVA and Bonferroni post -test. (**I**) Percentage of Annexin positive cells indicative of apoptosis (mean ± standard deviation, *n* = 4) after 96 hours treatment with 100 nM 1,25D ± 100 μM 4MU. Bars annotated with different letters are significant at *p* < 0.05 by one-way ANOVA and Bonferroni post -test.

Given the effects of 1,25D3 on the HA pathway in murine mammary tumor cells and in models of mammary cell transformation, we questioned whether similar responses would be observed in human breast cancer cells. The human triple negative breast cancer (TNBC) cell line Hs578T highly expresses HAS2 and produces high molecular weight HA which drives cell survival and invasion [21]. We thus assessed VDR function and the ability of 1,25D3 to regulate the HA pathway in this cell line. VDR protein expression was detected in the nucleus of Hs578T cells, whereas HAS2 and HA (visualized with bHABP) co-localized at cell surface protrusions (Figure 9A). Consistent with the results from murine cells, western blots (Figure 9B) demonstrated that 1,25D3 (100 nM, 48 h) enhanced *VDR* expression while reducing *HAS2* expression in in Hs578T cells. 1,25D did not alter *HAS3*, *HYAL1* or *HYAL2* expression but significantly increased *HYAL3*, in Hs578T cells (Supplementary Figure 4). HAS1 was not detected in control or treated Hs578T cells (data not shown). VDR transcriptional activity was confirmed by RT-qPCR measurement of *CYP24A1* (a well characterized *Vdr* target gene), which was up-regulated > 800-fold after 1,25D3 treatment (Figure 9C). The change in HAS2 protein in 1,25D3 treated Hs578T cells corresponded to reduction in *HAS2* mRNA and translated to a decrease in secreted HA (Figure 9C). Hs578T cells also highly express the breast cancer stem cell marker CD44, which when activated by HA promotes survival [37]. Notably, 1,25D3 treatment reduced *CD44* expression (Figure 9C), induced morphological changes indicative of differentiation (Figure 9D) and reduced both culture growth (Figure 9E) and migration (Supplementary Figure 6). Treatment with HA inhibitor 4MU or the glutamine analog 6-diazo-5-oxo-l-norleucine (DON), which inhibits the hexosamine pathway and generation of UDP-hexoses, strongly reduced density of Hs578T cultures, confirming that Hs578T cell growth/survival is dependent on HA (Figure 9F). Flow cytometric determination of cell cycle, cell number and annexin positivity (Figure 9G–9I) indicated that 1,25D3 cooperated with 4MU to inhibit entry into mitosis, reduce cell viability and enhance apoptosis. Collectively, these data suggest that, like murine mammary tumor cells and HMLE model systems, 1,25D3 suppresses HAS2 and HA synthesis in association with growth inhibition of human TNBC cells.

### Clinical relevance of *HAS2* to breast cancer

**Figure 10:**
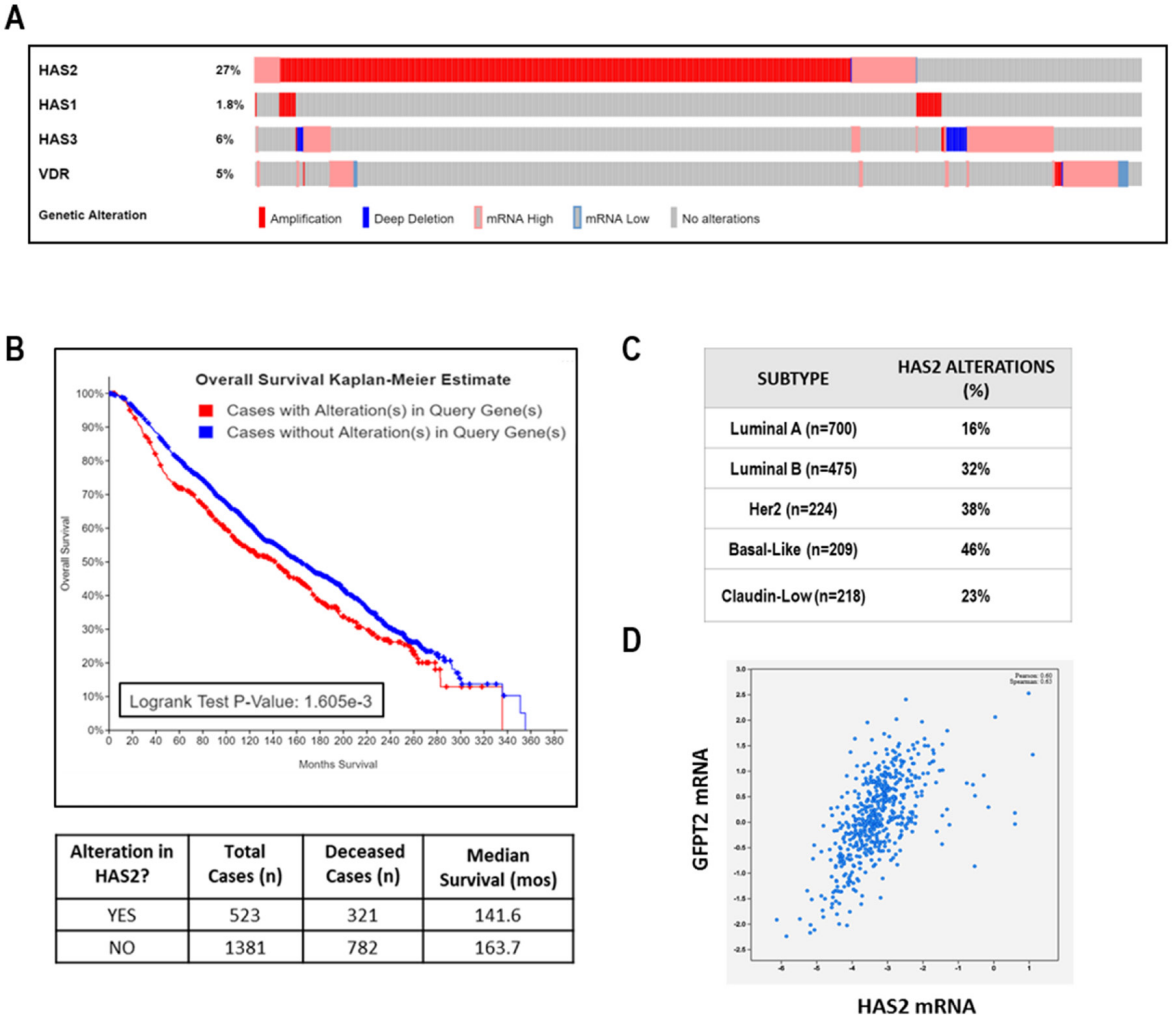
Clinical relevance of HAS2 in TCGA METABRIC dataset of human breast cancer. (**A**) Oncoprint showing genomic alterations in *VDR*, *HAS1*, *HAS2*, and *HAS3* in 1904 human breast tumors. Each column of light grey bars in this scheme represents one case of human breast cancer; colored bars indicate specific types of genomic alterations as indicated in the legend. (**B**) Kaplan Meier analysis of overall survival in METABRIC dataset stratified by *HAS2* alterations. (**C**) *HAS2* alterations according to breast cancer subtype. (**D**) Correlation between *HAS2* and *GFPT2* expression. All data were calculated from publicly accessible METABRIC database at cBio Cancer Genomics Portal.

The clinical relevance of *HAS2* in human breast cancer was assessed in the METABRIC dataset accessed via cBIO Portal [38, 39]. We annotated genomic alterations including mutations based on exome sequencing, copy number changes based on the Genomic Identification of Significant Targets in Cancer (GISTIC) algorithm and mRNA abundance based on RNA-Seq (Z score threshold ± 2). We found the overall frequency of genomic alterations in *HAS2* was 27% in 1904 cases of Breast Invasive Carcinoma with complete data (Figure 10A). Remarkably, all but two of the 523 tumor-associated changes in *HAS2* were amplifications or up-regulations suggesting that enhanced HA synthesis is frequent in breast cancer. Alterations in *HAS1* (< 2%) and *HAS3* (6%) were rare, suggesting that HAS2 is the most relevant HA synthase in breast tumors. Of note, genomic alterations in *VDR* were also rare (5% of cases) and the majority of these were amplifications or mRNA up-regulations, suggesting retention of vitamin D signaling in the majority of breast cancers. Kaplan–Meier analysis indicated that the presence of *HAS2* alterations in tumors correlated with significant reduction in median overall survival from 164 months to 143 months (Figure 10B). This finding is consistent with the observation that *HAS2* alterations were more common in the aggressive subtypes HER2+, Luminal B and Basal-Like (Figure 10C). Given that HAS2 activity depends on the availability of UDP-hexoses, it is not surprising that *GFPT2*, the rate limiting enzyme of the hexosamine pathway which is inhibited by DON (Figure 10D), was strongly associated with *HAS2* expression in this dataset (Figure 10D). Surprisingly, the association of *HAS2* alterations with survival was more pronounced in pre-menopausal women and in those whose tumors are estrogen receptor positive (Supplementary Figure 7) suggesting that HA production may identify a more aggressive subset of hormone dependent disease. Collectively, our studies indicate that HAS2 and GFPT2 represent attractive drug targets in human breast cancer.

## DISCUSSION

This work defines a novel role for 1,25D3 and the VDR in control of HA synthesis in epithelial tissues that likely contributes to its anti-cancer actions. We evaluated the relationship between 1,25D3, VDR, and HAS2 function in several model systems. In murine mammary tumor cells, we observed rapid and sustained down-regulation of *Has2* by 1,25D3. Notably, the decreased expression of *Has2* in VDR positive cells was sufficient to significantly reduce both cell-associated and secreted HA. More importantly, exogenous addition of high molecular weight HA (similar in size to that produced by HAS2) rescued VDR positive cells from 1,25D3 mediated growth inhibition. Low molecular weight HA, similar in size to fragments generated by hyaluronidase enzymes, was unable to rescue cells from 1,25D3 mediated growth inhibition. These data provide evidence that down-regulation of *Has2* leading to reduced synthesis of high molecular weight HA may be an important contributor to 1,25D3 mediated growth inhibition. Our data is consistent with reports that 1,25D and other endogenous vitamin D steroids inhibited TGF-β1-induced HAS2 expression and HA synthesis in association with anti-fibrotic actions, reduced HA accumulation in UV-treated mouse skin and decreased serum HA in a rat fibrosis model [40–42]. Furthermore, vitamin D supplementation of hepatitis patients (15,000 IU/week for 48 weeks) significantly reduced serum HA at all three time points studied (12, 24, and 48 weeks) [43]. Collectively, these data suggest that vitamin D comprehensively inhibits the HA pathway both *in vitro* and *in vivo*, supporting the translational relevance of our work.

We have also extended these findings in model systems of EMT and oncogenesis as well as in an established human TNBC cell line. We established that *HAS2* expression and HA production are elevated in normal human mammary epithelial cells induced to undergo EMT (through stable expression of TGFβ, SNAIL or TWIST) and in those expressing oncogenic H-RAS^V12^, indicating that deregulation of HA production may be an early and frequent event in breast tumorigenesis. 1,25D3 reduced HAS2 and HA secretion and acted additively with an HA synthesis inhibitor to slow growth of cells expressing TGFβ, SNAIL and TWIST. Similar results were observed in Hs578T cells, an aggressive TNBC cell line characterized by high HAS2 expression and production of high molecular weight HA [21]. VDR expression and transcriptional activity was confirmed in Hs578T cells, as 1,25D3 treatment induced the canonical VDR target gene *CYP24A1* over 800-fold. In Hs578T cells, 1,25D3 also reduced expression of *HAS2* and *CD44* and decreased HA secretion in association with phenotypic evidence of differentiation, inhibition of cell cycle progression and induction of apoptosis.

Using cells derived from VDRKO mice, we established that *Vdr* was necessary and sufficient for regulation of *Has2* by 1,25D3. No changes in *Has2* expression, HA secretion, or cell density were observed in response to 1,25D3 treatment of KO240 cells which were derived from VDRKO mice [1]. However, when KO240 cells were engineered to express human VDR (KO^hVDR^ cells), all of these responses were triggered by 1,25D3. These data suggest that both murine and human VDR are capable of repressing *Has2* when bound by 1,25D3. It is worth noting that 1,25D3 did not suppress *HAS2* in mammary epithelial cells expressing H-RAS^V12^, likely because this oncogene has consistently been shown to corrupt VDR signaling [34–36]. Collectively these data establish that 1,25D3 regulation of *Has2* is mediated through VDR rather than through alternative receptors such as the retinoic acid receptor-related orphan receptors (RORs) which can be activated by other dihydroxyvitamin D3 metabolites including 20,23(OH)_2_D_3_ [44].

Although the regulation of *HAS2* by 1,25D3 is dependent on VDR expression, no consensus VDREs have been identified in its promoter region by *in silico* analysis [45]. A potential alternative mechanism for this down-regulation is through the actions of microRNAs. Interestingly, miR-498, which has been shown to contain a consensus VDRE in its 5′ regulatory region, can target both *HAS2* and *CD44* [46]. Additional studies will be necessary to determine whether miR-498 or other microRNAs contribute to down-regulation of *HAS2* by 1,25D3.

Previous studies have shown that VDRKO mice are more susceptible to DMBA-induced breast cancer than WT mice [30]. HA interacts with several receptors including CD44 and RHAMM, leading to activation of signaling cascades that promote cell migration and proliferation [47]. Here we have demonstrated that *Vdr* ablation leads to increased HAS2 protein and accumulation of HA in normal tissues and in tumors. Since forced expression of HAS2 in MMTV-Neu mice enhances tumorigenesis [19], it is plausible that the HA accumulation observed in VDRKO mice contributes to their increased sensitivity to carcinogen induced tumors, however further studies would be needed to directly address this possibility.

To assess the translational relevance of our studies we interrogated the METABRIC TCGA dataset of > 1900 cases of human breast cancer. Surprisingly, few human breast tumors (5%) displayed genomic alterations in *VDR*, and most of the alterations that were detected were amplifications. Although *VDR* may frequently be retained in breast tumors, many women with breast cancer are vitamin D deficient [48, 49]. Therefore, attention to vitamin D status and/or vitamin D supplementation may be necessary to ensure appropriate VDR transcriptional activity in tumors. In support of a beneficial effect of vitamin D in cancer, the most recent meta-analysis of vitamin D trials including VITAL [50] indicated a significant reduction in cancer mortality with vitamin D supplementation. However, additional research is needed to determine the patient subsets most likely to benefit from attention to vitamin D status. Our data suggest that the anti-tumor actions of vitamin D may be mediated in part through suppression of HA signaling. In the METABRIC dataset, *HAS2* was overexpressed in 27% of all breast tumors, with highest frequency in those subtypes with poor prognosis (Luminal B, HER2+ and Basal-like). Kaplan–Meier curves of overall survival indicate significantly reduced median survival of patients whose tumors highly expressed *HAS2*. In this dataset, we also found a strong positive correlation between *HAS2* and *GFPT2*, suggesting that up-regulation of the hexosamine metabolic pathway may be necessary to generate UDP-hexoses for incorporation into HA by HAS2. If so, this metabolic vulnerability could be targeted with drugs that block GFPT2 activity. Since HA content is increased during breast cancer progression and elevations in *HAS2* and HA correlate with poor prognosis, further studies to evaluate the association of vitamin D signaling and the HA pathway in aggressive human breast cancers are of significant interest. Our data suggest that combining vitamin D supplementation with therapies that target HA signaling (i.e., CD44) or the hexosamine pathway (GFPT2) may be therapeutically beneficial.

## MATERIALS AND METHODS

### Cellular model systems

Murine mammary tumor cells with differential VDR expression (WT145 and KO240) were generated in our lab from wild-type and VDRKO mice [1]. The KO^hVDR^ cells were KO240 cells engineered to stably express human VDR (hVDR), the clone used here was KO^hVDR-4^ as described in LaPorta and Welsh [4]. All murine cell lines were maintained in DMEM/F12 media (50:50) supplemented with 5% charcoal-stripped FBS [1]. Fibroblasts isolated from DMBA-induced tumors of WT and VDRKO mice by collagenase digestion were adapted to culture and maintained in DMEM/F12 media (50:50) supplemented with 5% charcoal-stripped FBS. The effects of EMT were examined in engineered immortalized human mammary epithelial (HMLE) cells stably expressing GFP (control), SNAIL, TWIST or TGFβ (obtained from Dr. Sendurai Mani, MD Anderson Cancer Center, TX, USA) which were maintained in DMEM/F12/Media 171 (1:1) with mammary epithelial growth supplements (MEGS), insulin (5 ug/mL), EGF (5 ng/mL) and hydrocortisone (0.25 mg/mL) [32]. The effect of oncogenic transformation was evaluated in HMLE cells stably expressing H-RAS^V12^ (obtained from Dr. Robert Weinberg, MIT, MA, USA) which were maintained in Media 171 with MEGS [51]. The established human TNBC cell line Hs578T was obtained from ATCC and cultured in high-glucose DMEM with HEPES, 10% FBS and human insulin (10 ug/mL). All cell lines were maintained in a 37°C and 5% CO_2_ incubator with antibiotics and passaged every 3–4 days.

### Culture density, cell cycle, viability, and apoptosis assays

Culture density was assessed in cells plated in 24-well plates and treated the following day with indicated treatments or vehicle (ethanol for 1,25D3; methanol for 4MU; H_2_O or PBS for DON). After 96 hours, cells were fixed with 1% glutaraldehyde in PBS, stained with 0.1% crystal violet and air-dried overnight. The following day, stain was resuspended in 0.2% Triton X-100 and absorbance was read at 590 nm as an indicator of adherent cell density. Treatments included 1,25D3, 4MU, DON or vehicles as indicated in figure legends (all from Sigma). Low (29 kDa) and High (1.54 × 10^6^ kDa) molecular weight HA (R&D Systems, Minneapolis, MN) were suspended in H_2_O and added to media at a final concentration of 500 μg/mL. Cell cycle, viability and annexin positivity was assessed on a Millipore MUSE cell analyzer using Muse^®^ Count and Viability kit, Cell Cycle kit and Annexin V & Dead Cell Kit according to manufacturer’s directions.

### Reverse-Transcriptase quantitative PCR (RT-qPCR)

Cells were typically treated with 100 nM 1,25D3 or vehicle for 24 hours, but some experiments involved extended time courses as indicated in the figure legends. mRNA was isolated with the RNeasy Mini Kit (Qiagen) according to manufacturer’s instructions and used for cDNA synthesis. RT-qPCR was performed for *HAS1*, *HAS2*, *HAS3*, *HYAL1*, *HYAL2*, *HYAL3*, *VDR*, *CYP24A1*, and *CD44* gene expression using primers from Origene and SYBR green master mix (Life Technologies Applied Biosystems). Fold change was calculated as ΔΔC_t_ after normalizing to 18S and expressed relative to the ΔΔC_t_ of vehicle treated cells (for 1,25D3 treatments) or parental cell lines (for EMT and H-RAS^V12^ model systems).

### Analysis of HA

Secreted HA was quantitated by solid-phase sandwich ELISA of conditioned media removed after treatment (1,25D3, 4MU or respective vehicles). Cells were trypsinized and counted after the last time point for normalization. The low molecular weight (15–40 kDa), medium molecular weight (75–350 kDa), and high molecular weight (> 950 kDa) forms of HA are equally detected in this assay. Briefly, Nunc Maxisorp 96-well Immunoplates (ThermoFisher Scientific) were coated with HABP (non-biotinylated; Millipore) overnight at 4°C. Next day, the plate was washed with PBS containing 0.1% Tween 20 (PBST) and blocked with PBST containing 2% BSA for 1 hour at 37°C. After another series of washes, samples and HA standards (10 to 320 ng/mL; R&D Systems, Minneapolis, MN, USA) were added and incubated for 1 hour at 37°C. After another series of washes, bHABP was added and incubated for 1 hour at 37°C. After washing to remove unbound bHABP, and incubation with streptavidin-HRP (R&D Systems) for 1 hour at 37°C, TMB peroxidase substrate (SureBlue TMB; SeaCare Life Sciences, Milford, MA, USA) was added. Reactions were stopped with 1 M HCl and absorbance was measured at 450 nm with a background correction at 590 nm on a Victor^3^ V microtiter plate reader (PerkinElmer, Waltham, MA, USA). The amount of HA extrapolated from the standard curves was corrected for media blanks and dilution factors and expressed as absolute values in total media (for time courses) or per 10^6^ cells (for end point measurements). For particle exclusion assays to detect cell surface HA [27], cells were treated with 100 nM 1,25D3 for 48 hours prior to removal of media, PBS washes and addition of glutaraldehyde stabilized sheep erythrocytes (Fitzgerald Industries International, Acton, MA, USA). Images were obtained after erythrocytes settled on the plate and quantitation of the excluded areas was on an INCell 2200 high content cell analyzer.

### Western blotting

Whole cell lysates from cells treated for 48 hours with 100 nM 1,25D3 or EtOH were separated on SDS-PAGE gels, transferred to PVDF, and probed with mouse monoclonal VDR antibody (D6, Santa Cruz) or goat polyclonal HAS2 (Y-14) antibody (Santa Cruz). Blots were developed with ECL Plus substrate (Pierce) for 10 minutes and processed on a Storm 860 Molecular Imaging System using the 450 nm (blue) light source.

### Immunofluorescence

WT145 and KO^hVDR^ cells were seeded into 4-well chamber slides at a density of 20,000 cells/well and treated the next day with 100 nM 1,25D3 or vehicle. 48 hours after treatment, slides were fixed and permeabilized with ice cold methanol for 10 minutes, rinsed with PBS, and blocked with Dako Protein Block for 1 hour at room temperature. Slides were stained with rabbit polyclonal HAS2 antibody (H-60, SantaCruz Biotechnology) overnight at 4°C. After washing with PBS, slides were incubated with AlexaFluor 488 secondary antibody (Life Technologies), washed and coverslipped with ProLong Gold with DAPI (Invitrogen). Hs578T cells were seeded in chamber slides, fixed in ice cold methanol and immunostained for HAS2 (4E7, Abcam), VDR (D6, Santa Cruz) or HA (bHABP). After primary antibody incubations, slides were incubated with appropriate secondary antibodies (Life Technologies) and coverslipped with ProLong Gold with DAPI (Invitrogen). Imaging was performed on a Leica DMI6000 microscope with a TCS SP5 confocal laser scanner using Leica Application Suite AF version 2.6.0.7266 software or on a Zeiss Axioskop 2 microscope equipped with Axiovision software.

### Tissue analyses

In a previous study [52], inguinal mammary glands and dorsal skin were harvested from female WT and VDRKO mice maintained on a high calcium “rescue” diet and treated with DMBA. Xenografts of WT145 and KO240 cells grown in nude mice supplemented with estrogen were available from another previous study [1]. These published studies were conducted at the University of Notre Dame under IACUC-approved protocols. Archived samples, which had been formalin fixed, were sectioned at 5μ and interrogated for abundance of mucins, HAS2, and HA. Alcian blue was used as a general stain for acidic mucins which include HA. Slides were deparaffinized, rehydrated and stained with alcian blue (pH 2.5) for 5 minutes. For detection of HAS2 or HA, slides were deparaffinized and rehydrated as above prior to quenching of endogeneous peroxidase activity with 3% hydrogen peroxide. Non-specific binding was blocked with the Avidin/Biotin blocking kit (Vector Labs) followed by Dako protein block. For HA, slides were incubated with bHABP (Calbiochem) overnight at 4°C, rinsed with PBS, developed with the VECTASTAIN Elite ABC kit (Vector Labs) and detected with DAB chromogen as per manufacturer’s directions. For HAS2, slides were sequentially incubated with rabbit polyclonal HAS2 (H-60, Santa Cruz Biotechnology) and biotinylated secondary antibody prior to development with the VECTASTAIN Elite ABC kit as for HABP. For all stains, slides were counterstained with hematoxylin, rinsed, dehydrated and mounted with Permount. Images were acquired on a Zeiss Axioskop 2 microscope equipped with Axiovision software.

### Statistical analyses

GraphPad Prism software (La Jolla, CA, USA) was used for statistical analysis. Significant outliers as identified with the Grubbs test were removed. Significance was determined by Student’s *t* test (for 2 group comparisons) or one-way ANOVA (for 3 or more group comparisons) Post-hoc testing was with either Dunnetts or Bonferroni analysis as indicated in figure legends. Differences between means were considered significant when *p* < 0.05.

## SUPPLEMENTARY MATERIALS



## Abbreviations

1,25D3: 1,25-dihydroxyvitamin D3
4MU: 4-methylumbelliferone
bHABP: biotinylated HA binding protein
DAB: 3,3′-Diaminobenzidine
DON: 6-diazo-5-oxo-l-norleucine
ELISA: enzyme linked immunosorbent assay
EMT: epithelial mesenchymal transition
GISTIC: Genomic Identification of Significant Targets in Cancer algorithm
HA: hyaluronic acid
HABP: HA binding protein
HAS 1, 2, 3: Hyaluronan synthases
HMLE: immortalized human mammary epithelial cell line
HMW: high molecular weight
H-RAS^V12^: oncogenic form of H-RAS
HYAL: hyaluronidase
KO: knockout
KO240: cell line derived from DMBA mammary tumor induced in VDRKO mice
KO^hVDR^: KO240 cell line engineered for stable expression of human VDR
LMW: low molecular weight
MW: molecular weight
RT-PCR: reverse transcriptase polymerase chain reaction
TNBC: triple negative breast cancer
VDR: vitamin D receptor
WT: wild type
WT145: cell line derived from DMBA mammary tumor induced in WT mice
