# Supplementary material for: Inhibition of HAS2 and hyaluronic acid production by 1,25-Dihydroxyvitamin D3 in breast cancer
Source: Oncotarget. 2020 Jul 28;11(30):2889–905. doi: 10.18632/oncotarget.27587 (PMC7392624; doi:10.18632/oncotarget.27587)
Supplement: Supplementary file 1 [file oncotarget-11-2889-s001.pdf]

Inhibition of HAS2 and hyaluronic acid production by 1,25-Dihydroxyvitamin D_3 in breast cancer

SUPPLEMENTARY MATERIALS

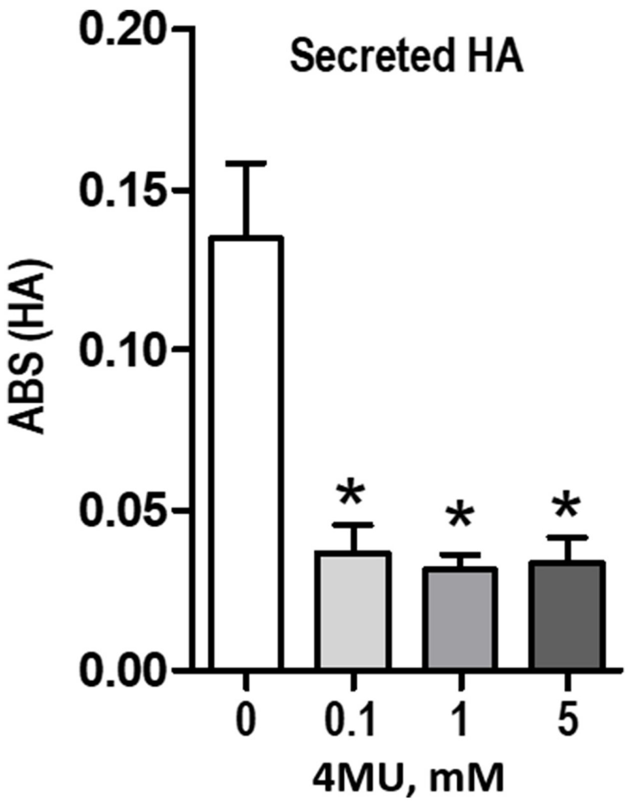

Supplementary Figure 1: Effect of 4MU on secreted HA in WT145 cells. HA was evaluated by ELISA in conditioned media of murine WT145 cells treated with up to 5 mM 4MU for 72 hours. $^{*}p < 0.05$ treated vs control samples, one-way ANOVA.

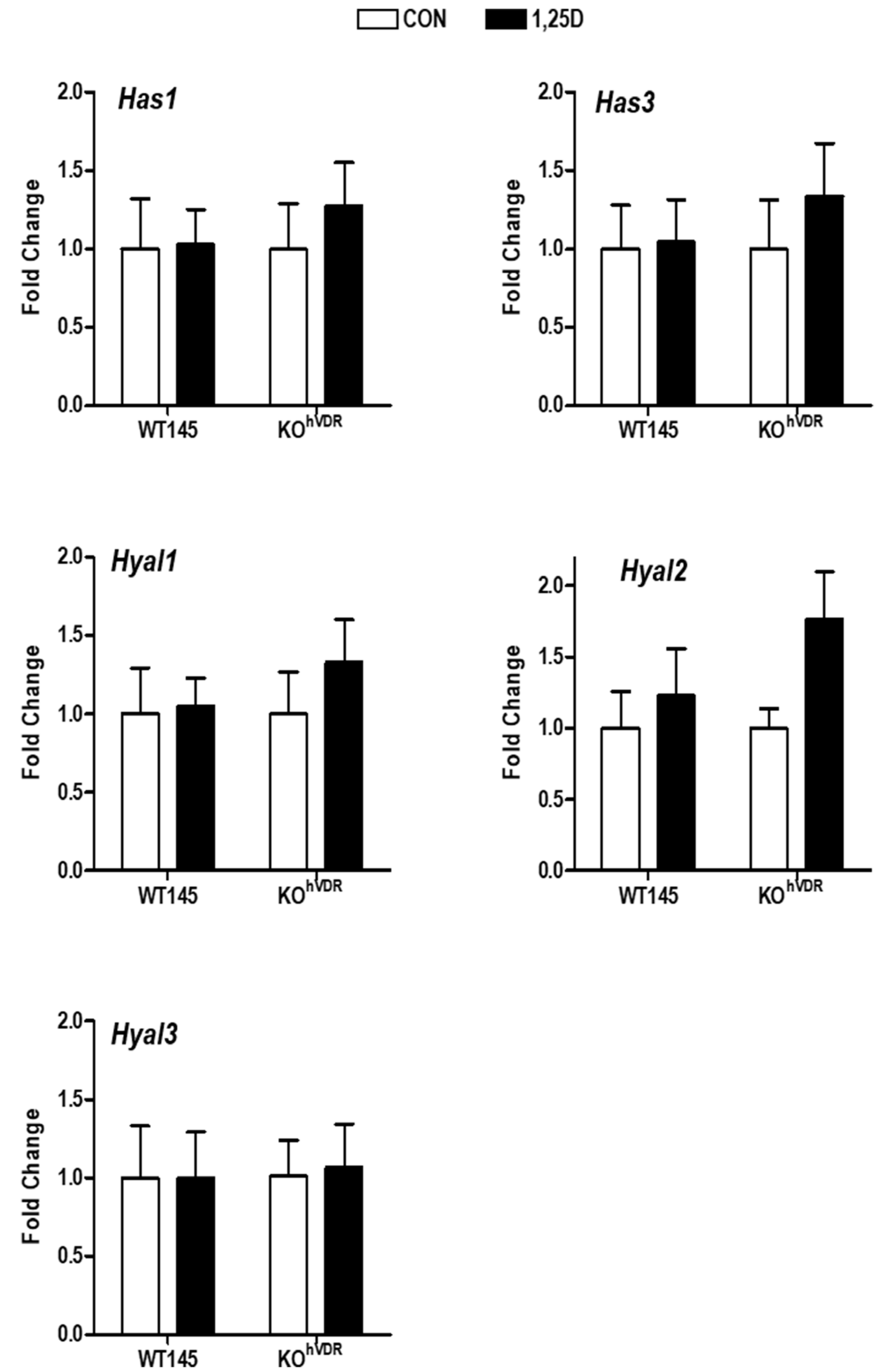

Supplementary Figure 2: Effects of 1,25D3 on *HAS1*, *HAS3* and *HYAL* gene expression in WT145 and KO^{hVDR} cells. Cells were treated with 100 nM 1,25D3 or vehicle for 24 h prior to RT-qPCR assessment of mRNA expression. Data were normalized against GAPDH with vehicle treated samples for each cell line set to 1. Each bar represents mean ± standard deviation of 3 independent samples analyzed in duplicate. No statistically significant differences.

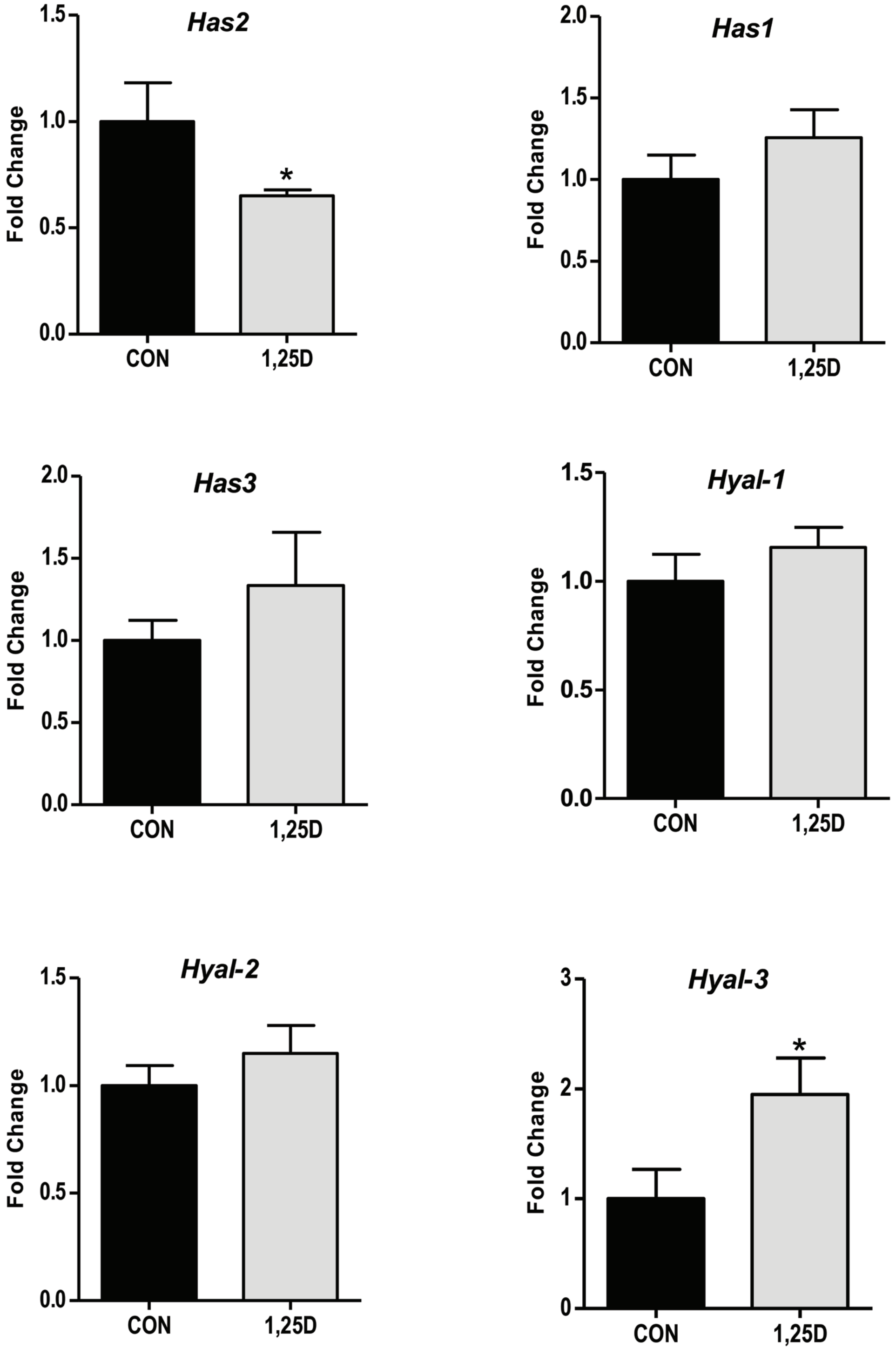

Supplementary Figure 3: HA pathway gene regulation by 1,25D3 in fibroblasts derived from tumors of WT mice. Cells were treated with 100nM 1,25D3 or vehicle for 24 h prior to RT-qPCR assessment of mRNA expression. Data was normalized to 18S RNA and expressed relative to untreated control cells. Each bar represents mean ± standard deviation of 3 independent samples analyzed in duplicate. $^{*}p < .05$, control vs treated, Student's *t* test.

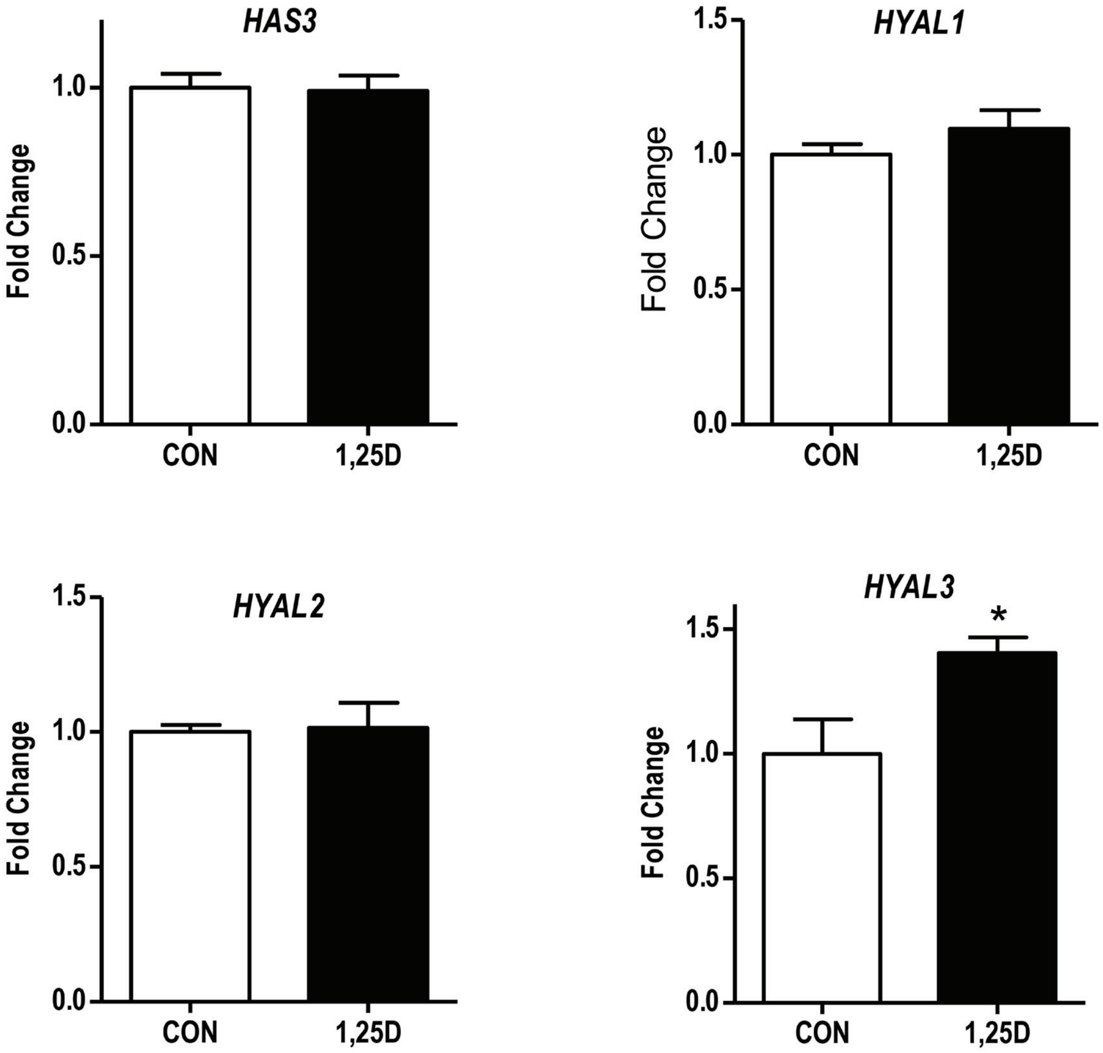

Supplementary Figure 4: Effect of 1,25D3 on *HAS3* and *HYAL* gene expression in Hs578T cells. Cells were treated with 100 nM 1,25D3 or vehicle for 24 h prior to RT-qPCR assessment of mRNA expression. Data was normalized to 18S RNA and expressed relative to untreated control cells. Each bar represents mean ± standard deviation of 3 independent samples analyzed in duplicate. $^{*}p < .05$, control vs treated, Student's *t* test.

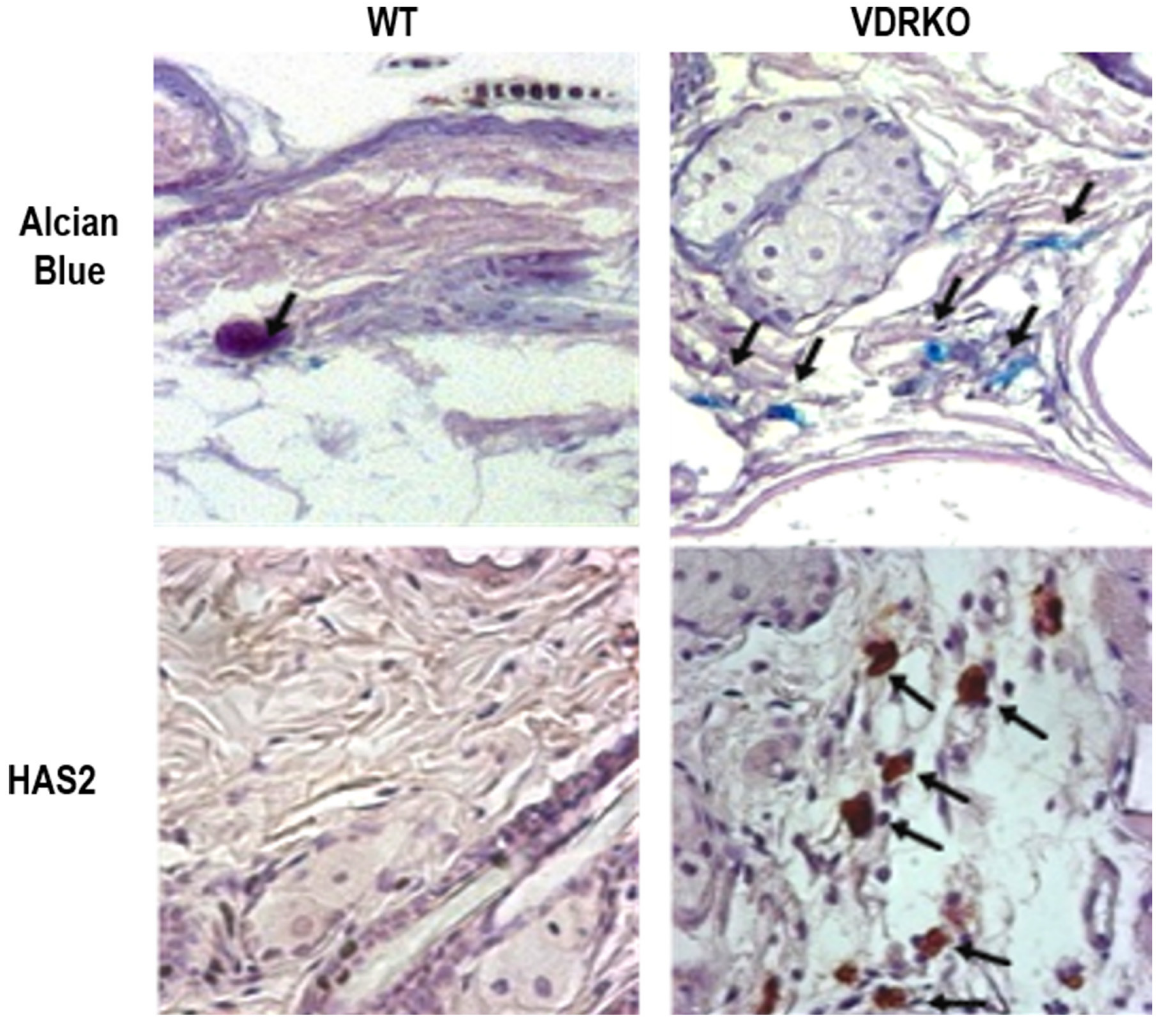

Supplementary Figure 5: Alcian Blue staining and HAS2 immunoreactivity in epidermis of WT and VDRKO mice. Archived epidermal tissue from Zinser et al. [31] was processed for alcian blue staining of glycosaminoglycans and HAS2 immunochemistry. Arrows indicate positive cells.

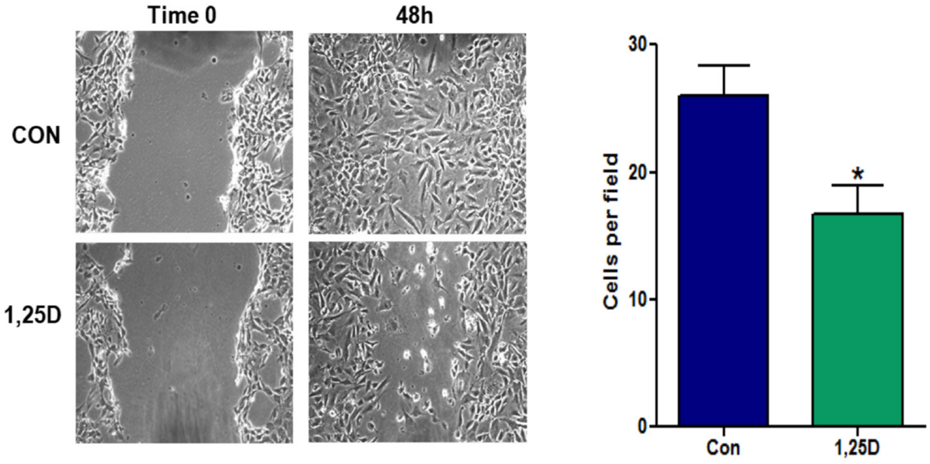

Supplementary Figure 6: Effects of 1,25D3 on migration of Hs578T cultures. Cells in 6 well plates were pre-treated with 100 nM 1,25D3 for 24 h. At confluence, wells were uniformly scratched and imaged every 15 mins using an EVOS live cell imaging system. Representative images for the same fields at 0 and 48 hours are shown. After 24 h, cells remaining in the scratched area were counted. Data are mean ± standard error. $^{*}p < 0.05$, Student's *t* test.

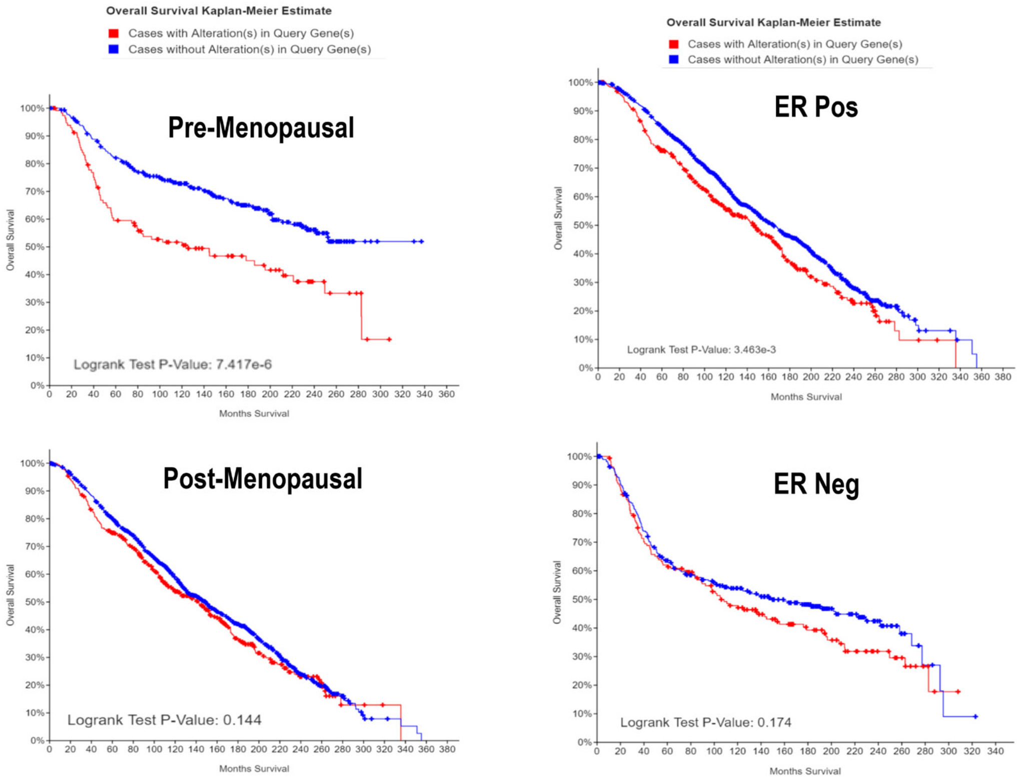

Supplementary Figure 7: Impact of *HAS2* gene alterations on survival of patients stratified by menopausal status and tumor estrogen receptor positivity (measured by IHC). Kaplan-Meier analyses indicated that *HAS2* gene alterations were more predictive of overall survival in pre-menopausal women and in those whose tumors were estrogen receptor positive. Calculated from the Metabric TCGA dataset at cBIO Portal.
